# Uncovering Genes and Ploidy Involved in the High Diversity in Root Hair Density, Length and Response to Local Scarce Phosphate in *Arabidopsis thaliana*

**DOI:** 10.1371/journal.pone.0120604

**Published:** 2015-03-17

**Authors:** Markus G. Stetter, Karl Schmid, Uwe Ludewig

**Affiliations:** 1 Institute of Crop Science, Nutritional Crop Physiology, University of Hohenheim, Fruwirthstr. 20, 70593 Stuttgart, Germany; 2 Institute of Plant Breeding, Seed Science and Population Genetics University of Hohenheim, Fruwirthstr. 21, 70593 Stuttgart, Germany

## Abstract

Plant root hairs increase the root surface to enhance the uptake of sparingly soluble and immobile nutrients, such as the essential nutrient phosphorus, from the soil. Here, root hair traits and the response to scarce local phosphorus concentration were studied in 166 accessions of *Arabidopsis thaliana* using split plates. Root hair density and length were correlated, but highly variable among accessions. Surprisingly, the well-known increase in root hair density under low phosphorus was mostly restricted to genotypes that had less and shorter root hairs under P sufficient conditions. By contrast, several accessions with dense and long root hairs even had lower hair density or shorter hairs in local scarce phosphorus. Furthermore, accessions with whole-genome duplications developed more dense but phosphorus-insensitive root hairs. The impact of genome duplication on root hair density was confirmed by comparing tetraploid accessions with their diploid ancestors. Genome-wide association mapping identified candidate genes potentially involved in root hair responses tp scarce local phosphate. K*nock-*out mutants in identified candidate genes (*CYR1*, *At1g32360* and *RLP48*) were isolated and differences in root hair traits in the mutants were confirmed. The large diversity in root hair traits among accessions and the diverse response when local phosphorus is scarce is a rich resource for further functional analyses.

## Introduction

Phosphorus (P) is an essential macronutrient, which is involved in several crucial processes in the plant. Nevertheless, phosphorus is one of the most limiting resources in global crop production and the high quality global reserves are likely depleted within a century [1]. The low mobility of P in the soil requires a local response of plants to counteract any deficiency and plant roots extend their contact surface just behind the root tip with root hairs to increase the water and nutrient uptake [2]. Their importance for phosphorus supply under low availability has been proven using *Arabidopsis thaliana* [3] and barley mutants [4] that lack root hairs.

A large number of root hair and non-root hair-specific genes and proteins are known and many genes involved in their development have been identified. A large gene and protein network exclusively expressed in either root hair or non-root hair cells, of which many are related to the specific growth pattern of the hairs, has been identified [5,6]. In addition to genetic factors, hormonal signals and environmental factors, such as nutrient (especially phosphorus) deficiencies, control root hair development [7,8]. Among plant hormones, auxin and ethylene [9–11] and as was discovered more recently, strigolactones [12], appear to have the largest impact on root hair length and density.

In *A*. *thaliana*, as well as in many other plants, the low availability of phosphorus, manganese or iron increases the root hair density [13,14]. Phosphorus deficiency leads to local and systemic transcriptional responses [15], and plants respond with a complex gene network to cope with lower phosphate supply [16]. Root hairs in *Arabidopsis* are normally only formed at positions where a rhizodermal cell is in contact with two cortical cells. The restricted longitudinal elongation of rhizodermal cells and ectopic root hairs contribute to the higher density of root hairs under P starvation in *A*. *thaliana* [17].

The root hair length depends on the duration of the tip growth phase after they had been initiated [7,8]. Therefore, it is likely that different genes control the responses of root hair density and root hair length. Several genes, including transcription factors, have been associated with altered root hair density and length under P deficiency. Mutants in *bHLH23 (At3g25710)* increased root hair formation in low P [18], mutants in *PHL1 (At5g29000)* and *PHR1 (At4g28610)* affected root hair length under low P [19]. Furthermore, loss of the SUMO E3 ligase gene *SIZ1 (At5g60410)* [20], loss of *HSP2 (At5g03730)*, a Raf-like kinase [21], or the loss of the F-box gene *FBX2 (At5g21040)* [22] all led to higher root hair density in low P. By contrast, the loss of the ubiquitin protease 14 gene, *UBP14 (At3g20630)*, was associated with inhibition of root hair growth under low P [23]. Furthermore, regulators of the cell fate, such as *ENHANCER OF TRY AND CPC1 (ETC1)* are also involved in regulating root hair density under P deficiency [17]. Understanding the complex mechanisms of root hair acclimation to nutrient stress is of great importance to use limited nutrient resources more efficiently.

Complex and plastic traits, such as root hair acclimation, require a reproducible experimental set-up and system of measurement. Early phenotypes of seedlings can be significantly influenced by the stored nutrient reserves of the seed. We noted that when germinated on uniform—P plates, several accessions did not show homogenous seedling and/or root growth. Therefore, we developed a setup in which roots grew from a fully supplied medium (to ensure sufficient initial nutrient supply) into a medium without P. This plate assay allowed the measurement of large numbers of root hairs. We analyzed root hair traits in a large population of natural *A*. *thaliana* accessions from a wide geographic distribution to identify the diversity of root hair traits and their responses to the scarce local P supply.

## Materials and Methods

### Plant material

A panel of 166 diverse *Arabidopsis thaliana* (L.) Heynh accessions was used. These were chosen for high geographical diversity (S1 Table) and the availability of detailed genomic data. Genotypes were divided into six main populations (Central Europe, Northern Europe, Iberian Peninsula, Mediterranean, Central Asia and North America) and three small populations (Cape Verde, Canary Islands and Japan) reflecting their geographic origin (S1 Fig.). All seeds were derived from plants grown in nutrient-rich garden soil (Einheitserde EET, Einheitserde- und Humuswerke, Sinntal-Jossa, Germany), to ensure sufficiently high P content in all seeds. *D*iploid Wa-1 (2x) and tetraploid Col-0 (4x) seeds [24] were from D. Chao (China). Knock-out mutants *rlp48* (N677688), *aox1d* (N692539), *at1g32360–1* (N671169) *and cyr1* (N667777) were obtained from the *Arabidopsis* stock center (Nottingham, GB) and homozygosity and absence of the relevant transcript were checked by standard methods [25].

### Split plates and growth conditions

Vertically placed split squared Petri dishes (120 mm x 120 mm x 17 mm, Greiner bio-one, Kremmünster, Austria) with a top compartment containing P (1 mM KH_2_PO_4_) and a lower test compartment without P were used. The growth medium was composed of 5 mM KNO_3_, 2 mM MgSO_4_, 2 mM Ca(NO_3_)_2_, 70 μM HBO_3_, 14 μM MnCl_2_, 1 μM ZnSO_4_, 0.5 μM CuSO_4_, 10 μM NaCl, 0.2 μM Na_2_MoO_4_, 40 μM FeEDTA, 4.7 mM MES and 43 mM sucrose, pH was adjusted to 5.7 with 1 M KOH. The medium solidified with 1.2% phytoagar. All plates were prepared as split plates with a solid acrylic glass barrier of approximately 13 cm width and 3 cm height with two 1.5 cm deep notches allowing the fixation of the barrier inside the 12 cm wide Petri dish (S2 Fig.). Medium containing P was first added to the top part. Once solidified, the barrier was removed and the empty part was filled with medium containing either P or not, where KH_2_PO_4_ was substituted by 1 mM KCl. *Arabidopsis* seeds were surface sterilized with 70% ethanol and 0.05% TritonX 100 for 15 minutes under continuous shaking and were washed twice with 70% ethanol for 5 minutes. After washing, the seeds were dried on filter paper under a clean bench.

Eight to fifteen seeds were placed 1 cm above the split. To enhance and synchronize germination, plates were stored for two days at 4°C in darkness, before being kept under 21°C and continuous light for 12 hours. After light treatment, plates were kept in cold and darkness for another day before being transferred into the growth chamber. Plants were grown under constant light (180 μE) and 21°C until the root had a length of 3 cm, usually after 10 to 20 days.

### Experimental design

The experiment with the diverse panel of accessions was conducted as incomplete split-plot design with sub-sampling, as growth chamber space was limited. Three accessions (*Col-0*, *Sha* and *Copac-1*) were analyzed as standards in all eight incomplete blocks and behaved highly similar. A split-plot design was used for the polyploids and the *knock-out* mutants.

### Image acquisition and analyses

For each accession images of six to ten roots per treatment were taken with 20x magnification with a Zeiss AxioCam MRm (Carl Zeiss, Jena, Germany) monochrome camera under a Zeiss Stemi 2000-c binocular microscope (Carl Zeiss, Jena, Germany) when roots had reached 3 cm of length to ensure an equal developmental stage. While for each mutant 10–15 roots per treatment were measured. Images were acquired 1 cm above the root tip, where root hairs were fully developed, as the length of the root tip greatly differed between accessions.

Root images of ca. 5 mm length were analyzed with ImageJ software (http://imagej.nih.gov). Images were subdivided into 1 mm segments with the *Grid* plugin for ImageJ (rsbweb.nih.gov/ij/plugins/grid.html). Root hair length was determined by measuring five straight hairs in focus per root on alternating sides. Root hair density was determined by counting all visible root hairs in a 1 mm segment in the center of the image.

### Statistical data analysis

A mixed linear model was used for data analysis, reflecting the experimental design. The following model was employed for the diversity study:

Y= GEN x TRT + REP: ((REP/BLK) x ROW x COL)/PLATE)/PLANT

Where,

Y = Response variable (root hair density or root hair length).

GEN = Fixed effect of the genotype

TRT = Fixed effect of the treatment

REP = Fixed effect of the replicate

BLK = Random effect of the incomplete block

COL = Random effect of the column position in the growth chamber

ROW = Random effect of the row position in the growth chamber

PLATE = Random effect of the Petri dish

PLANT = Residual effect of the plant

x = main effects + interaction

/ = nested relationship

For root hair length, the mean over five measurements per root was calculated. Data analysis was performed with the MIXED procedure in SAS/STAT of SAS 9.3. Variance homogenity was reviewed and outliers were identified and removed based on studentized residuals. Values with studentized residuals larger than 4 were removed from the dataset. Mean values and standard error (SE) are given.

The root hair surface integrates both traits, length and density. The surface area is proportional to the “surface density” and was calculated by applying a cylinder model with a root hair diameter of 0.01 mm:

Root hair surface density = hair length * hair diameter * π * hair density

### Genomic data preparation

For all accessions, either whole-genome sequence data from the ‘1001Genomes Project’ [26] or high-density single nucleotide polymorphism (SNP) genotypes from the RegMap project [27] were publicly available. These data-sets were combined into a single set of SNP-markers, using all SNPs that were called in all three data-sets and had less than 30% missing data in the sequences and a minor allele frequency >0.05. Missing data were imputed using MaCH [28]. As a result, a total of 160640 SNPs were used for further analysis.

### Genome-wide association (GWA) mapping

Mapping was performed with a multi-locus mixed model (MLMM) to account for the structured population and improve the power of the study [29]. The kinship was calculated with GAPIT based on the whole marker set [30]. Further, adjusted means of the different traits were used as phenotypic input. The GWA mapping approach was applied to seven traits: root hair density and length under control and scarce local phosphate supply conditions, and normalized (relative) root hair density, length and surface response in scarce local phosphate supply.

The chromosomal regions around SNPs with Bonferroni corrected p-values smaller than 0.0001 were analyzed for nearby genes based on the TAIR10 gene annotation (www.arabidopsis.org). Genes located in or near significant SNPs were identified with the online tool GBrowse [31] using a window size of 10 kb. Genes closest to the associated SNP were selected for further studies.

## Results

### Establishment of a root hair screening system for the acclimation to low local phosphorus

**Fig 1:**
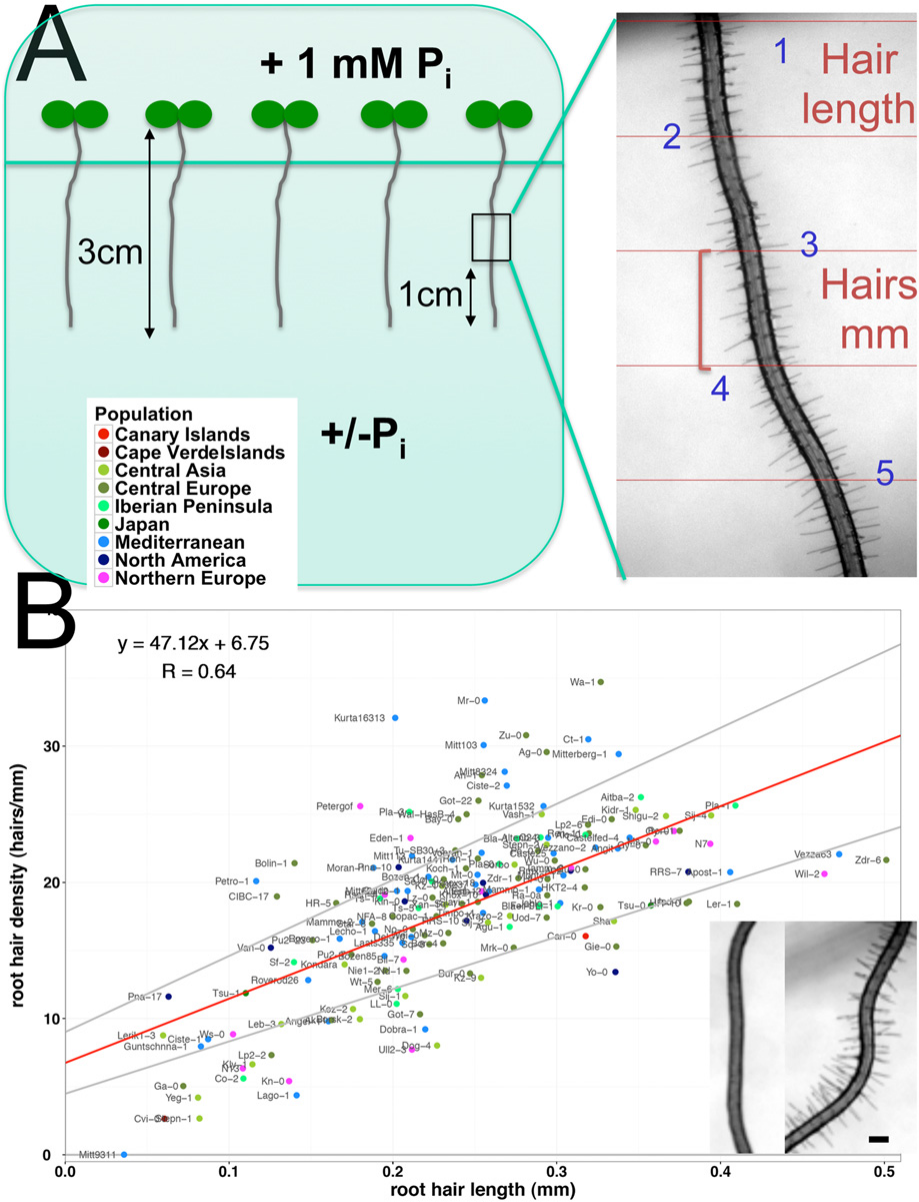
Natural variation in root hairs of 166 *Arabidopsis thaliana* accessions. (A) Split-plate assay. Right panel: Col-0 root in—P. Red lines are drawn 1 mm apart (B) Correlation (R = 0.64) and 95% confidence interval of root hair density and root hair length under control conditions. Inset: phenotype of Mitt9311 (left) and Wa-1 (right) under control conditions, scaling bar 0.1 mm. Values represent mean of replicates with each 5 plants.

To ensure similar conditions for germination and early seedling development of a large array of *Arabidopsis* accessions, seeds were germinated on the top compartment of split agar plates containing all nutrients, including phosphorus. Plates were placed vertically so that roots grew into the lower compartment. The lower compartment either contained phosphorus (+P) or did not contain phosphorus (-P) (Fig. 1A). The root hair parameters measured from these seedlings were highly reproducible in eight repetitions of blind assays, with little variation for individual genotypes. Overall, the broad sense heritability was 0.85 for the hair density and 0.86 for the hair length. The upper part of the roots was in contact with medium containing phosphorus and the lower part used for root hair scoring was in direct contact with the medium lacking P. This experimental design ensured that exclusively local responses of the hairs to scarce P (by elongation or increased density) were scored.

### Root hair diversity and response to scarce local phosphorus

**Fig 2:**
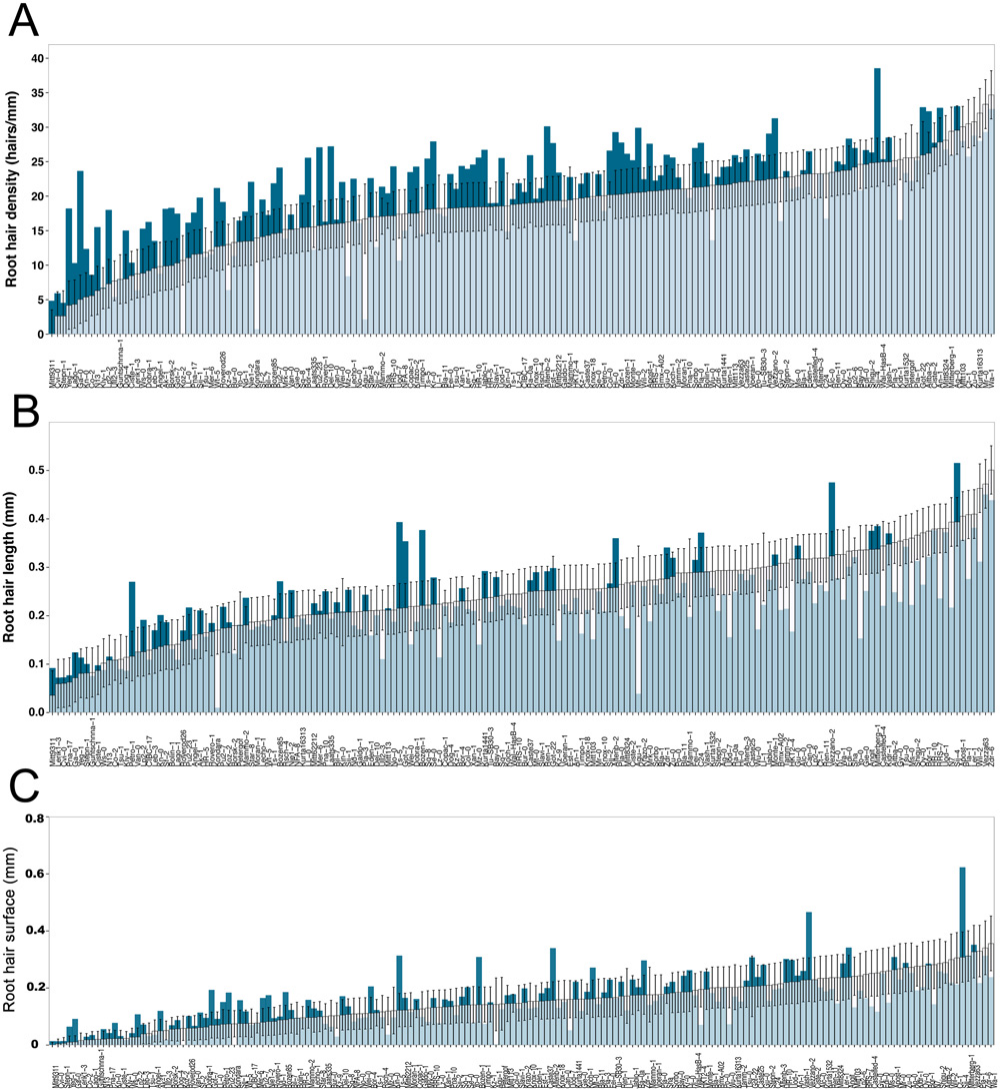
Natural variation in root hair traits of 166 *Arabidopsis thaliana* accessions. (A) Root hair density, (B) length, (C) surface under control (white bars) and scarce local phosphorus (blue bars). Error bars indicate standard error.

While some accessions developed almost no root hairs, others had over 30 hairs/mm (Fig. 1B). Col-0 had an average density (20.22 hairs/mm ± SE = 1.84) (Figs. 1B and 2A). A correlation between root hair density and length was observed (Fig. 1B). In Fig. 2A, bars indicate the root hair density from all accessions ordered from low to high density (white / light blue bars). The hair densities in scarce P are overlaid in the same figure with dark blue bars. Similar to the root hair density, accessions displayed high variation in root hair length (Fig. 2B). Observed lengths ranged from under 0.1 mm to almost 0.5 mm in the extreme cases (Figs. 1B, 2B, S2 Table). Col-0 did neither display outstandingly long nor short root hairs, similar as with its root hair density. Although root hair density and length were correlated in most accessions, some accessions showed very dense, but short hairs. By contrast, others showed few, but long root hairs (Figs. 1B, 2).

The root hair surface was calculated to combine the two measured parameters and to analyze the area available for nutrient uptake. By combining root hair density and length for each accession in this manner, root hair surface was a powerful measure to differentiate between accessions. The root hair surface area, calculated as a surface density that is proportional to the surface area, ranged from close to zero up to 0.4 mm, while under P starvation, accessions showed up to 0.6 mm (Fig. 2C).

Without local phosphate supply, the overall mean density (20.78 hairs/mm) was moderately increased compared to control conditions (18.34 hairs/mm). Nevertheless, the root hair density of accessions differed largely, ranging from 0.07 hairs/mm (Koz-2;SE = 4.88) up to 38.51 hairs/mm (Sij-4;SE = 3.49). Col-0 significantly increased the root hair density to 26.55 hairs/mm (SE = 1.85, p< 0.05) in scarce local P supply. 75 accessions increased the root hair density without local phosphate supply, whereas 72 showed similar densities as under control conditions, and 19 accessions even showed a decreased root hair density (Fig. 2A).

**Fig 3:**
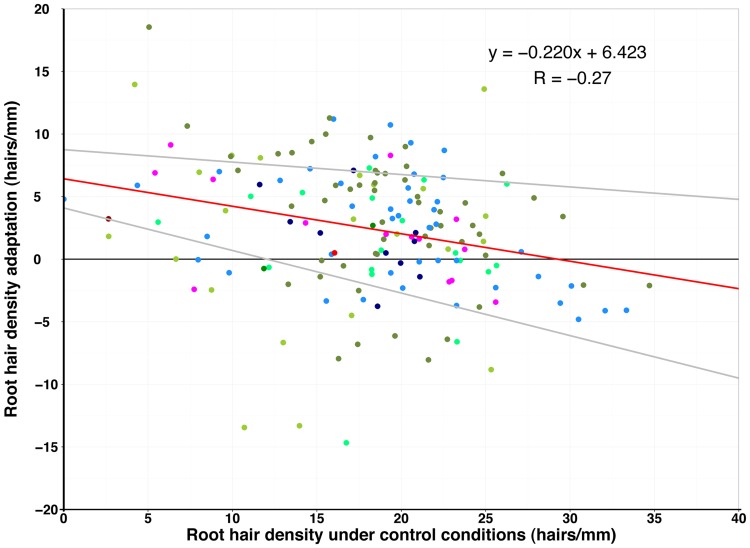
Dependence of root hair density change with—P on root hair density under control conditions. Weak negative correlation (R = -0.27, red line) and 95% confidence interval (grey lines) of root hair density response and density under control conditions.

Generally, in accessions with few root hairs, root hair density increased in scarce local P, while accessions with a high density under control conditions often did not respond to scarce P (Figs. 2A & 3). The root hair density in—P was thus weakly negatively correlated to the root hair density under control conditions (Fig. 3). Unlike overall mean hair density, mean hair length did not increase under local scarce P. Nonetheless, the response of root hair length to scarce P of accessions was diverse as well and changes in both directions were observed (Fig. 2B). Surprisingly, 41 accessions reduced root hair length under local scarce P in the growth medium (Fig. 2B).

**Fig 4:**
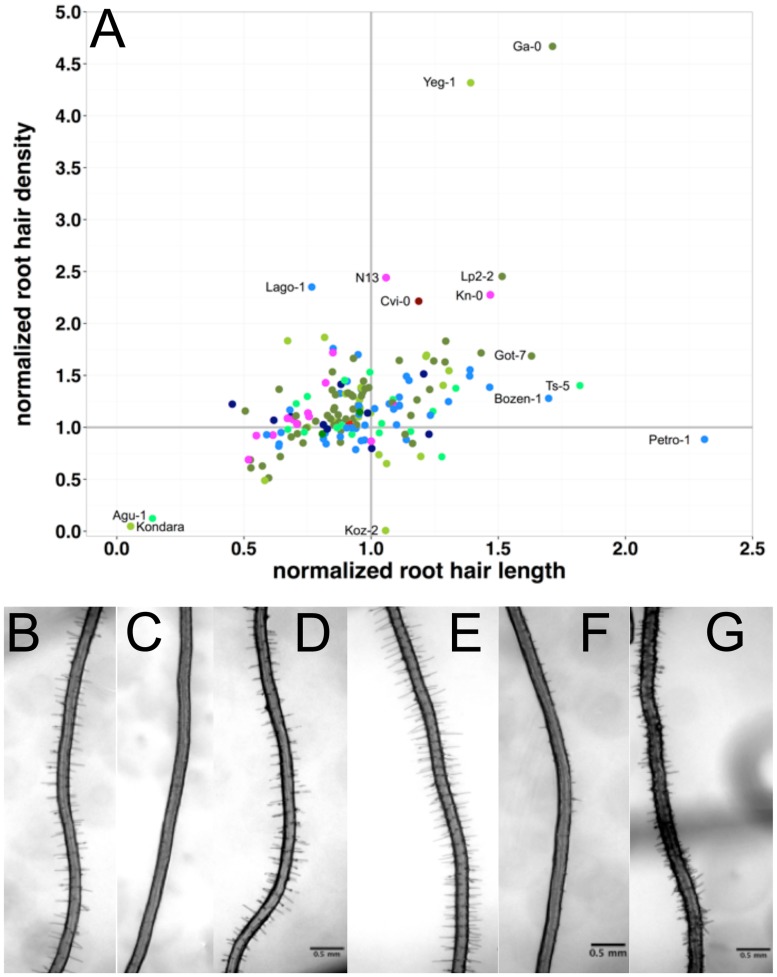
Root hair density and length responses to scarce local phosphorus (P) supply. The responses were normalized to the density and length under control conditions (+P) and are given as relative values under—P (values of 1 indicate no change). (A) Root hair density response (density-P / density +P) versus relative length (length-P / length +P). (B-G) root hair phenotypes of Kondara (B,C), Col-0 (D,E) and Ga-0 (F,G) under control and local scarce P. Scaling bars: 0.5 mm.

Some accessions displayed a very strong response to scarce local phosphate, particularly Agu-1 and Kondara (Fig. 2) reduced hair density and length by more than two-fold. By contrast, Pretro-1 increased hair length by more than two-fold, without changing the root hair density. On the other hand, Yeng-1 and Ga-0 (Figs. 2 & 4) increased hair density by almost five-fold; Lp2–2, N13, Cvi-0, Kn-0 and Lago-0 more than two-fold (Figs. 2 & 4). With regard to absolute values, Ga-0 displayed the largest increase of almost 20 hairs/mm under local P starvation. Other accessions increased root hair length even though they already had developed very long root hairs (e.g. Sij-4 and Vezzano-2; Figs. 2 & 4).

Regarding the sub-populations, there was no common pattern within these groups observed for root hair length, density and the response to scarce local phosphate supply. No geographic pattern explained differences in the root hair traits (Figs. 2, 3, 4).

### Genome-wide association mapping of root hair density and response to scarce local P

The phenotypic data was used for genome wide association (GWA) mapping. We used the multi-locus mixed model (MLMM) package [29], which is particularly suitable for structured populations as used in this study. Q-Q-plots showed that the observed p-values are close to the expectation (S3 Fig.).

**Fig 5:**
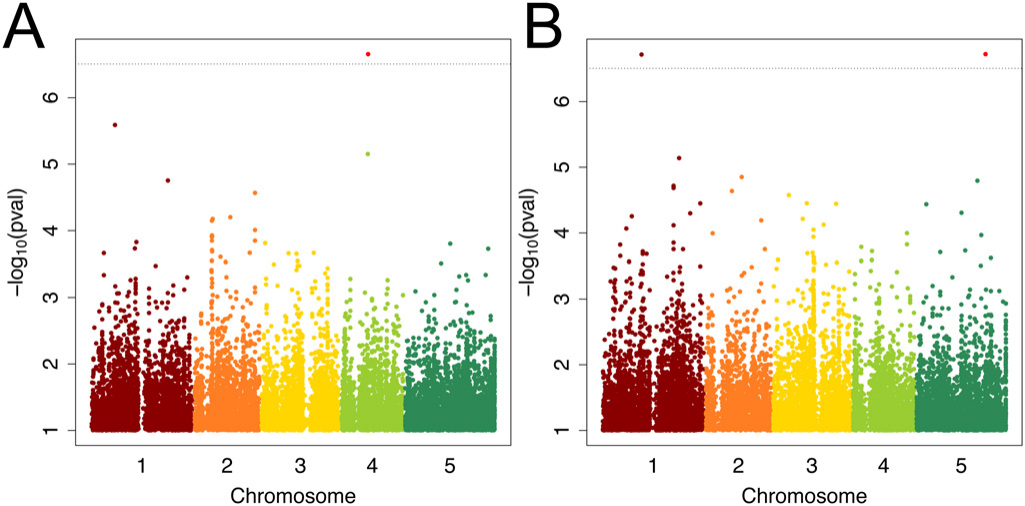
Manhattan-plots for root hair density (A) and root hair surface (B) response to local scarce phosphorus supply. Light red dots (above the significance threshold) indicate SNPs used as cofactors in MLMM. SNPs of different chromosomes are given in different colors. Horizontal line indicates significant Bonferroni corrected p-values at 5% significance level.

GWA mapping identified one highly significantly associated single nucleotide polymorphism (SNP) for root hair density in scarce local phosphate, being located 427 base pairs (bp) upstream to the Receptor Like Protein 48 (*At4G13880*; Fig. 5A, Table 1) on chromosome 4. For the combined trait root hair surface in scarce P, two significantly associated SNPs were identified (Fig. 5B, Table 1), one 3198 bp upstream of *CYTOKININ RESISTANT 1 (CYR1*, *At5G51230*), a polycomb gene involved in stress response and vegetative growth. The second significantly associated SNP is located between the gene coding for alternative oxidase 1D (*AOX1D*, *At1G32350*, 2045 bp upstream) and the uncharacterized CCCH-zinc finger protein gene *At1G32360* (Fig. 5B, Table 2, 2711 bp upstream). For other root hair traits, no significant SNPs were identified (S4 Fig., S5 Fig.).

**Table 1:** Candidate genes as detected for root hair density response to scarce local phosphate supply by genome-wide association study.

Chr	Pos[bp]	p-value	Gene	Position	Annotation
4	8025724	2.21E-07	*At4G13880*	upstream	*RLP48*, *receptor like protein 48*
1	7085727	2.57E-06	*At1G20440*	in gene	*COR47*, *dehydrin protein family*
4	7937687	7.03E-06	*At4G13640*	in gene	*UNE16*, *unfertilized embryo sac 16*
1	23016486	1.76E-05	*At1G62300*	down-stream	*WRKY6*, *transcription factor*
2	18286563	2.71E-05	*At2G44221*	in gene	*Protein of Unknown Function*
2	10855824	6.28E-05	*At2G25500*	upstream	*nosine triphosphate pyrophosphatase family protein*
2	5549098	6.68E-05	*At2G13370*	in gene	*CHR5*, *chromatin remodeling 5*
2	5317801	7.09E-05	*At2G12940*	upstream	*UNE4*, *unfertilized embryo sac 4*
2	18285063	9.80E-05	*At2G44220*	in gene	*Protein of Unknown Function*

**Table 2:** Candidate genes as detected for root hair surface response to scarce local phosphate supply by genome-wide association study.

Chr	Pos[bp]	p-value	Gene	Position	Annotation
5	20820955	1.89E-07	*At5G51230*	upstream	*CYR1*, *cytokinin resistant*
1	11670614	1.92E-07	*At1G32360*	upstream	*Zinc finger (CCCH-type) family protein*
1	11670614	1.92E-07	*At1G32350*	upstream	*AOX1D*, *alternative oxidase 1D*
1	22986142	7.24E-06	*At1G62200*	upstream	*PTR6*, *peptide transporter*
2	10855824	1.41E-05	*At2G25500*	upstream	*nosine triphosphate pyrophosphatase family protein*
5	18375271	1.60E-05	*At5G45340*	upstream	*CYP707A3*
1	21316400	1.91E-05	*At1G57560*	upstream	*MYB50*, *transcription factor*
1	21316982	2.07E-05	*At1G57560*	in gene	*MYB50*, *transcription factor*
2	7900874	2.30E-05	*At2G18160*	upstream	*ATBZIP2*, *transcription factor*
3	4768402	2.65E-05	*At3G14300*	in gene	*ATPME26*
3	10174453	3.53E-05	*At3G27480*	in gene	*Cysteine/Histidine-rich C1 domain family protein*
1	29355538	3.54E-05	*At1G78070*	in gene	*Transducin/WD40 repeat-like superfamily protein*
3	18987992	3.60E-05	*At3G51120*	in gene	*DNA binding;zinc ion binding;nucleic acid binding;nucleic acid binding*
5	2987972	3.65E-05	*At5G09640*	upstream	*SNG2*
5	13590082	4.93E-05	*At5G35370*	in gene	*S-locus lectin protein kinase family protein*
1	26328682	5.02E-05	*At1G69910*	upstream	*Protein kinase superfamily protein*
1	8740591	5.57E-05	*At1G24706*	upstream	*THO2*
3	8953457	6.11E-05	*At3G24540*	in gene	*PERK3*
2	16738390	6.43E-05	*At2G40090*	in gene	*ATH9*
3	15250210	7.48E-05	*At3G43302*	in gene	*gypsy-like retrotransposon family*
1	21315507	7.64E-05	*At1G57560*	upstream	*MYB50*
1	7085727	8.56E-05	*At1G20440*	in gene	*COR47*
3	12189492	8.91E-05	*At3G30620*	in gene	*gypsy-like retrotransposon family (Athila)*

Among the 70 genomes, for which the entire genome sequence was available, each of these genes was highly polymorphic. *RLP48* has 37 nucleotide differences within the coding region, of which 25 led to amino acid exchanges. For *CYR1*, a total of 9 SNPs alter the amino acid sequence, while for *At1G32360*, of 31 SNPs only 9 lead to amino acid differences among these 70 accessions.

### Confirmation of candidate genes involved in root hair traits and the response to scarce local P

**Fig 6:**
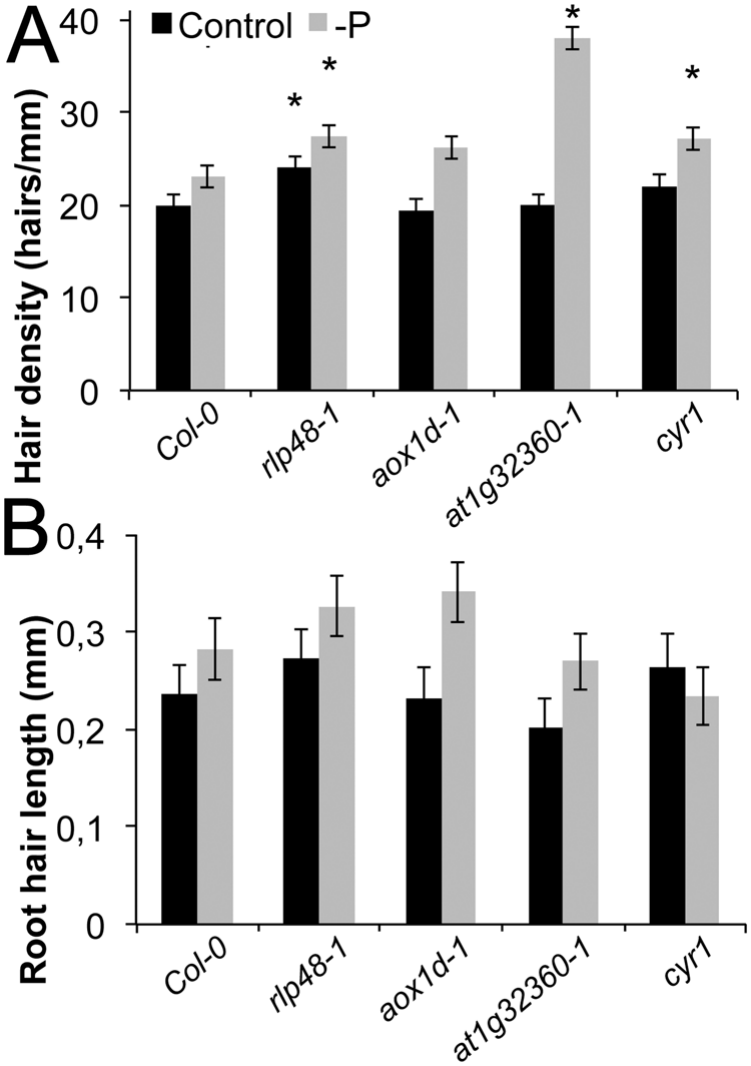
Root hair length and density in *loss of function* mutants of candidate genes. (A) Root hair density of wild type (Col-0), *rlp48–1*, *aox1d-1*, *at1g32360–1*, *cyr1* (all mutants in the Col-0 background). Black bars: with P, grey bars: without P. (B) Root hair length of wild type (Col-0), *rlp48–1*, *aox1d-1*, *at1g32360–1*, *cyr1*. Black bars: control with P, grey bars: without P, error bars indicate standard error. Statistically significant data points compared to diploid individuals under the same conditions are given as ‘*’ (p<0.05).

Root hair density, length and response to scarce P were subsequently analyzed in *loss-of-function* mutants of candidate genes that were within or neighboring the three significantly associated SNPs, to validate the biological function of the regions and genes found by GWA mapping. Homozygous *T-DNA* mutants were used for that purpose. A *loss-of-function* mutant of the candidate receptor like protein 48 (*rlp48–1)*, had higher root hair density in both conditions, consistent with the hypothesis that this gene is important for root hair density and its relation with P (Fig. 6A). However, the length of the root hairs in that line was not affected.

Furthermore, one associated SNP was between the genes encoding the alternative oxidase 1D (*AOX1D*, *At1G32350)* and a putative transcription factor gene (*At1G32360)*. While root hair length and density were not affected by the loss of the *AOX1D* gene, root hair density (and hence the surface) was markedly increased under scarce P in *at1g32360–1* (Fig. 6). Finally, a *loss-of-function* line *cyr1* of *CYTOKININ RESISTANT1* also showed a significantly higher root hair density in scarce local phosphate supply.

Taken together, the phenotypes of *loss-of-function* mutants were consistent with natural allelic variation of these genes contributing to natural variation of root hair traits. Interestingly, the loss of two identified candidate genes led to higher root hair densities under control conditions and P insensitivity, suggesting that the identified genes are P-independent repressors of root hair density. By contrast, the repressing effect *of At1g32360* appears to be—P dependent.

### Genome duplication affects root hair traits

**Fig 7:**
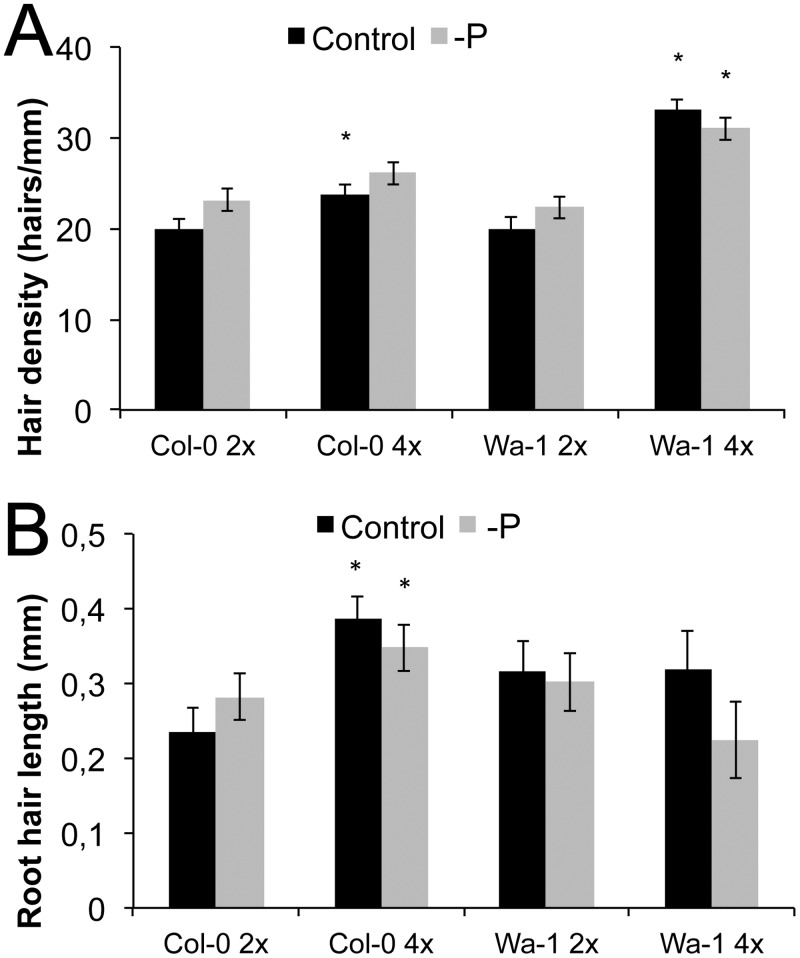
Root hair length and density in isogenic accessions differing in ploidy. (A) Root hair density of Col-0 (2x), Col-0 (4x), Wa-1 (2x), Wa-1 (4x), 2x, diploid; 4x, tetraploid, Black bars: with P, grey bars: without local P. (B) Root hair length of Col-0 (2x), Col-0 (4x), Wa-1 (2x), Wa-1 (4x). Black bars: with P, grey bars: without P. Statistically significant data points compared to diploid individuals are given as ‘*’(p<0.05), error bars indicate standard error.

The accession that maximally increased its surface by root hairs among all accessions and also had the highest hair density was Wa-1 (from Warsaw, Poland), an autotetraploid accession (Fig. 2). Interestingly, polyploids of *A*. *thaliana* had recently been identified to have higher potassium uptake and increased potassium in the shoot [24]. Another autotetraploid accession, Ciste-2 (from Cisterna di Latina, Italy), was also among the accessions with the highest root hair density (Fig. 2). In both cases, root hair density was not different in scarce local P, but hairs of Wa-1 were even slightly shorter without local phosphate supply (Figs. 2, 7). We further checked whether the ploidy level influences root hair density or length in the isogenic diploid *Wa-1* (2x) line, which was derived by haploid induction [32]. Although these lines were isogenic, root hairs were 65% less dense under control conditions, with the trend of increased density under scarce local P (Fig. 7), contrary to the tetraploid Wa-1 (4x) accession. Similarly, the Col-0 colchicine-doubled tetraploid line (Col-0, 4x) had 18% higher hair density under control conditions, but no significant density increase was seen for the response to scarce local phosphate supply. Furthermore, the Col-0 (4x) line had much longer root hairs already under control conditions (by 63%), but the length response to scarce local P was reversed compared to the P sufficient condition, similar to the response to P in Wa-1 (4x). This suggests that an increased chromosome set number strongly increased root hair surface on phosphate sufficient medium, but a further increase when local phosphate supply was scarce is not seen in such polyploids.

## Discussion

### Diversity of root hairs and response to scarce local phosphate supply

This study presents an overview of the diversity of root hairs and their response to scarce local P. We developed a robust high throughput root hair phenotyping assay that allowed the quantification of many individuals with reproducible results. Quantifying the two most important aspects of root hair response, hair length and density, this study revealed that a much higher diversity of root hair phenotypes is present in *Arabidopsis* than previously thought. Candidate genes for the response to locally scarce P were identified and confirmed by *loss-of-function* mutants, but these were not in loci previously identified in the response of root hairs to P deficiency. However, it must be stressed that in our approach, the roots were not systemically P-deficient since the top part of the plant had access to sufficient P. Genes previously identified in the acclimation to scarce P might respond to systemic low P and were thus not identified here. It is also possible that these genes are not sufficiently polymorphic in the panel of accessions used for the GWAS approach. Furthermore, comparisons with older studies clearly indicated that the root hair acclimation to low P appears to be different than the acclimation to scarce local P, which was investigated here. Our study is consistent with the suggestion that different genes control the global and the local response to insufficient P supply [16].

Overall, the strategies of acclimation of plant roots to scarce local P are diverse and do not only involve root hairs. As a response to low P, some plants alter root architecture to increase the lateral root abundance close to the soil surface, which, in combination with an increased root hair density, may be much more effective than changing each parameter alone [33]. Acclimated accessions that can be used for further studies of root hair responses were identified. These might be suitable parents for recombinant inbred lines for more accurate gene mapping.

In agreement with earlier studies,-P most frequently leads to a correlated increase of root hair density [3,7,34]. Nonetheless, some accessions not following this general pattern were identified, e.g., Kondara and Agu-1, which drastically decreased their root hair density under local scarce P supply. The combination of the two traits, density and length into a single trait, root surface, revealed an even stronger difference between the accessions (Figs. 1, 2 and 3).

An increase in the number and length of root hairs may be a suitable strategy for accessions with few, short root hairs under control conditions. Increase only the number of root hairs, but not their length, appears to be established in accessions that already have long hairs. Some accessions, by contrast, do not alter their root hair patterning under scarce local P at all. These accessions might not respond locally, or alter other root traits, for example by developing more lateral root branching or by changing their P metabolism. Furthermore, these accessions might already be acclimated to an environment with low P supply and do not sense any deficiency in P under our conditions; in these ecotypes, systemic signaling may indicate sufficient P in the plant shoot.

Surprisingly, some accessions even decreased the number and length of root hairs with scarce local phosphate supply. They may invest their resources into other root responses, such as the formation and growth of lateral roots into zones, where P is available. Such different strategies should be further investigated, to obtain a conclusive answer to the question if and how the accessions adjust under scarce local phosphate supply. Interestingly, there was no large-scale geographic pattern associated with the distribution of the accessions that responded negatively to scarce P. This suggests that this negative response may either reflect the response to local soil conditions, where increased root surface area that is commonly observed in P deficiency, is negatively associated with the reproduction success or that the root hairs have low impact in that respect (and are neutral with respect to evolution).

The root hair density was much more responding to the scarce local P than the root hair length. Other studies, in which P was deficient in the whole plant, described a root hair elongation of up to 3 times under low P conditions [3,7]. Besides, the absolute values of root hair density were lower in this study than described in previous work, despite that they were analyzed at a similar developing state. One reason for this may be that in previous studies the entire growth medium lacked P. Our results strongly suggest that at least some accessions can locally sense and respond to scarce P by changing root hair density, but also hair length, although the length was affected only in a few accessions.

Under-P, root hair length and root hair density were correlated in most accessions, in accordance with previous studies [3]. However, accessions with contrasting reactions to-P were also identified, and they may be interesting candidates to independently study the pathways of root hair density and length. The correlation between root hair length and density under control conditions and under scarce local P was also high for most accessions, showing that the response follows a general pattern (Fig. 3). Nevertheless, there are accessions with trait values outside the confidence interval of this correlation. These are suitable for generating a genetically diverse mapping population for mapping the underlying genes more accurately.

It had been shown that if the primary root tip comes in contact with a P-deficient medium, primary root growth is reduced [35]. This effect then shortened the total root length, either by reduction in longitudinal epidermal cell length or less numbers of epidermal cells. Each mechanism may increase the root hair density and it will be interesting to test these possibilities for all 166 accessions studied. Whether a lower root hair density is a consequence of sensing P by root hair founder cells, or just a secondary effect of sensing scarce local P in the root tip is beyond the scope of the study. To avoid interference with different root elongation, here all hairs were quantified at the same root length. Similarly, iron toxicity has been associated with the reduced primary root length under P deficiency, while the same (100 μM Fe-EDTA) iron concentrations were non-toxic in the presence of P [36]. The plates lacking P in the lower compartment used here contained iron at non-toxic (40 μM Fe-EDTA) levels [36].

### Candidates from genome-wide association mapping

GWA mapping with MLMM yielded significantly associated SNPs for several traits. MLMM was developed to perform well with structured populations, which is clearly the case with sub-populations used here (Segura et al., 2012). For instance, we included closely related alpine accessions, as well as closely related central Asian accessions (S1 Fig.). The GWA study with MLMM corrected for population structure and yielded significant SNPs, which were located in genes or in close proximity. We assumed that the closest genes to these SNPs are the best candidates, although it had been shown that causal SNPs are not necessarily closer linked to the trait in GWAS or more significant than nearby non-causal SNPs, since SNPs in such a small region are linked [37]. One significant SNP was close to receptor like protein 48 gene and a mutant (*rlp48–1)* lacking that gene showed higher root hair density in both conditions, consistent with the hypothesis that this gene plays a role in root hair development (Fig. 6). Furthermore, one SNP close to a putative transcription factor gene (*At1G32360)* was identified, and the importance of that gene for hair density was confirmed using a *loss-of function* mutant (Fig. 6). Another gene identified was *CYR1 (At5G51230*), a cytokinin-regulated gene that is involved in root growth. *Knock-out* plants of *CYR1* are cytokinin insensitive and develop shorter roots, but more and longer root hairs [38]. The *at1g32360–1* and *cyr1* mutants showed significantly higher root hair density than wild type only under—P condition, displaying a P-deficiency-hypersensitive phenotype. But none of them showed significant difference in root hair length from the wild type in P sufficient medium, suggesting that *At1g32360* and *CYR1* are involved in root hair density regulation upon P-deficiency. The coding region of each of these genes is polymorphic among 70 fully sequenced accessions, with several amino acid differences predicted from the sequence. However, causal SNPs may also be found outside the coding region, regulating the gene expression level.

### Ploidy matters for root hair length, density and acclimation to scarce P

The accession with the highest surface increase by root hairs in response to the scarce local P, and that with the highest hair density, was Wa-1, an autotetraploid accession (Fig. 2). We therefore also checked whether the ploidy level matters for root hair density and responses to P. Hairs of diploid Wa-1 were of similar length in—P and +P, but shorter in the tetraploid in scarce local P (Fig. 7). Tetraploid Col-0 (Col-0, 4x) showed longer and denser root hairs compared to the isogenic diploid line. In both cases polyploidy led to an increase in root hair density in sufficient local P, but scarce local P did not further increase the density or length, probably because root hairs were already maximally elongated and dense.

Polyploidy was found to influence other physiological traits in *A*. *thaliana*. For example, the genome duplication of tetraploids caused higher shoot K^+^ concentrations and salt resistance in various *A*. *thaliana* accessions [24]. Interestingly, the ploidy level of the root was exclusively responsible for this observation, as shown by grafting experiments [24]. In our study, two autotetraploids developed very long root hairs (Wa-1 and Ciste-2), but did not increase their length in response to low local P. Interestingly, the isogenic diploid Wa-1 (2x) line had less dense hairs, which increased in density with local scarce P. Similarly, compared to the diploid Col-0, which responded modestly to scarce local P, the tetraploid line had long, dense hairs that were little influenced by P (Col-0, 4x, Fig. 7). As these accessions are isogenic and only differ in their ploidy level, this indicates a strong impact of ploidy on root hair parameters (Fig. 7). The effect of the genome duplications on root hair parameters may also partially explain root K^+^ uptake and higher shoot K^+^ concentrations, as root hairs play a central role in K^+^ acquisition, at least under a low K^+^ supply [39].

## Conclusions

To our knowledge, this study presents the largest overview on root hair responses to local scarce P. The surprising high level of natural variation in the root hair length and density raises several questions with respect to its role in ecological acclimation to different environments. The developed screening method allows the quantification of many individuals. Using these measurements together with high SNP density for GWA mapping, genes and ploidy involved in the response of root hairs to scarce local P were revealed. It will be interesting to study how the identified candidate genes are involved in cellular responses and other plant strategies to cope with scarce P.

## Supporting Information

S1 Table List of *Arabidopsis* accessions used in this study and assigned population name. (DOC)

S2 Table Root hair density and length data. (XLSX)

S1 Fig Geographic origin of genotypes used for phenotyping. Different colors indicate regional sub-populations.
(TIF)

S2 Fig Split plate manufacture. The barrier in the plates was removed after solidification of the top compartment.
(TIF)

S3 Fig Q-Q plot of MLMM bias for sub-population corrections. First order (black dots) and second order (red dots) corrections for observed and expected distribution due to population bias.
(TIF)

S4 Fig Manhattan-plots for root hair density under control conditions (left) and root hair length (middle) and surface (right) under control conditions. SNPs of different chromosomes are given in different colors.
(TIF)

S5 Fig Manhattan-plots for root hair density with local scarce phosphorus supply (left), root hair length in scarce P (middle) and length response to local scarce phosphorus supply (right). SNPs of different chromosomes are given in different colors.
(TIF)

## References

[1] Cordell D, Drangert J-O, White S. The story of phosphorus: Global food security and food for thought. Glob Environ Chang. 2009; 19: 292–305.

[2] Datta S, Kim CM, Pernas M, Pires ND, Proust H, Tam T, et al Root hairs: development, growth and evolution at the plant-soil interface. Plant and Soil. 2011; 346: 1–14.

[3] Bates TR, Lynch JP. Root hairs confer a competitive advantage under low phosphorus availability. Plant Soil. 2001; 236: 243–250.

[4] Gahoonia TS, Nielsen NE. Phosphorus (P) uptake and growth of a root hairless barley mutant (*bald root barley*, *brb*) and wild type in low- and high-P soils. Plant Cell Environ. 2003; 26: 1759–1766.

[5] Lan P, Li W, Lin WD, Santi S, Schmidt W. Mapping gene activity of *Arabidopsis* root hairs. Genome Biol. 2013; 14: R67 10.1186/gb-2013-14-6-r67 23800126 PMC3707065

[6] Deal RB, Henikoff S. A simple method for gene expression and chromatin profiling of individual cell types within a tissue. Dev Cell. 2010; 18: 1030–1040. 10.1016/j.devcel.2010.05.013 20627084 PMC2905389

[7] Müller M, Schmidt W. Environmentally induced plasticity of root hair development in *Arabidopsis* . Plant Physiol. 2004; 134: 409–419. 14730071 10.1104/pp.103.029066 PMC371035

[8] Jones VA, Dolan L. The evolution of root hairs and rhizoids. Ann Bot. 2012; 110: 205–212. 10.1093/aob/mcs136 22730024 PMC3394659

[9] Rahman A, Hosokawa S, Oono Y, Amakawa T, Goto N, Tsurumi S. Auxin and ethylene response interactions during *Arabidopsis* root hair development dissected by auxin influx modulators. Plant Physiol. 2002; 130: 1908–1917. 12481073 10.1104/pp.010546 PMC166701

[10] Pitts RJ, Cernac A, Estelle M. Auxin and ethylene promote root hair elongation in *Arabidopsis* . Plant J. 1998; 16: 553–560. 10036773 10.1046/j.1365-313x.1998.00321.x

[11] Masucci JD, Schiefelbein JW. The *rhd6* Mutation of *Arabidopsis thaliana* Alters Root-Hair Initiation through an Auxin- and Ethylene-Associated Process. Plant Physiol. 1994; 106: 1335–1346. 12232412 10.1104/pp.106.4.1335 PMC159671

[12] Kapulnik Y, Resnick N, Mayzlish-Gati E, Kaplan Y, Wininger S, Hershenhorn J, et al Strigolactones interact with ethylene and auxin in regulating root-hair elongation in *Arabidopsis* . J Exp Bot. 2011; 62: 2915–2924. 10.1093/jxb/erq464 21307387

[13] Ma Z, Bielenberg DG, Brown KM, Lynch JP. Regulation of root hair density by phosphorus availability in *Arabidopsis thaliana* . Plant Cell Environ. 2001; 24: 459–467.

[14] Foehse D, Jungk A. Influence of phosphate and nitrate supply on root hair formation of rape, spinach and tomato plants. Plant Soil. 1983; 74: 359–368.

[15] Thibaud MC, Arrighi JF, Bayle V, Chiarenza S, Creff A, Bustos R, et al Dissection of local and systemic transcriptional responses to phosphate starvation in Arabidopsis. Plant J. 2010; 64: 775–789. 10.1111/j.1365-313X.2010.04375.x 21105925

[16] Chiou TJ, Lin SI. Signaling network in sensing phosphate availability in plants. Annu Rev Plant Biol. 2011; 62: 185–206. 10.1146/annurev-arplant-042110-103849 21370979

[17] Savage N, Yang TJ, Chen CY, Lin KL, Monk NA, Schmidt W. Positional signaling and expression of ENHANCER OF TRY AND CPC1 are tuned to increase root hair density in response to phosphate deficiency in Arabidopsis thaliana. PLoS One. 2013; 8: e75452 10.1371/journal.pone.0075452 24130712 PMC3794009

[18] Chen ZH, Nimmo GA, Jenkins GI, Nimmo HG. *BHLH32* modulates several biochemical and morphological processes that respond to Pi starvation in *Arabidopsis* . Biochem J. 2007; 405: 191–198. 17376028 10.1042/BJ20070102 PMC1925254

[19] Bustos R, Castrillo G, Linhares F, Puga MI, Rubio V, Perez-Perez J, et al A central regulatory system largely controls transcriptional activation and repression responses to phosphate starvation in *Arabidopsis* . PLoS Genet. 2010; 6: e1001102 10.1371/journal.pgen.1001102 20838596 PMC2936532

[20] Miura K, Lee J, Gong Q, Ma S, Jin JB, Yoo CY, et al SIZ1 regulation of phosphate starvation-induced root architecture remodeling involves the control of auxin accumulation. Plant Physiol. 2011; 155: 1000–1012. 10.1104/pp.110.165191 21156857 PMC3032448

[21] Lei M, Zhu C, Liu Y, Karthikeyan AS, Bressan RA, Raghothama KG, et al Ethylene signalling is involved in regulation of phosphate starvation-induced gene expression and production of acid phosphatases and anthocyanin in *Arabidopsis* . New Phytol. 2011; 189. 10.1111/j.1469-8137.2010.03555.x 21118263

[22] Chen ZH, Jenkins GI, Nimmo HG. Identification of an F-box protein that negatively regulates P(i) starvation responses. Plant Cell Physiol. 2008; 49: 1902–1906. 10.1093/pcp/pcn157 18930958

[23] Li WF, Perry PJ, Prafulla NN, Schmidt W. Ubiquitin-specific protease 14 (UBP14) is involved in root responses to phosphate deficiency in *Arabidopsis* . Mol Plant. 2010; 3: 212–223. 10.1093/mp/ssp086 19969521

[24] Chao DY, Dilkes B, Luo H, Douglas A, Yakubova E, Lahner B, et al Polyploids exhibit higher potassium uptake and salinity tolerance in *Arabidopsis* . Science. 2013; 341: 658–659. 10.1126/science.1240561 23887874 PMC4018534

[25] Yang H, Postel S, Kemmerling B, Ludewig U. Altered growth and improved resistance of *Arabidopsis* against *Pseudomonas syringae* by overexpression of the basic amino acid transporter AtCAT1. Plant, Cell and Environment. 2014; 37: 1404–1414. 24895758 10.1111/pce.12244

[26] Weigel D, Mott R. The 1001 genomes project for *Arabidopsis thaliana* . Genome Biol. 2009; 10: 107 10.1186/gb-2009-10-5-107 19519932 PMC2718507

[27] Horton MW, Hancock AM, Huang YS, Toomajian C, Atwell S, Auton A, et al Genome-wide patterns of genetic variation in worldwide *Arabidopsis thaliana* accessions from the RegMap panel. Nat Genet. 2012; 44: 212–216. 10.1038/ng.1042 22231484 PMC3267885

[28] Li Y, Willer CJ, Ding J, Scheet P, Abecasis GR. MaCH: using sequence and genotype data to estimate haplotypes and unobserved genotypes. Gen Epidemiol. 2010; 34: 816–834. 10.1002/gepi.20533 PMC3175618 21058334

[29] Segura V, Vilhjalmsson BJ, Platt A, Korte A, Seren U, Long Q, et al An efficient multi-locus mixed-model approach for genome-wide association studies in structured populations. Nat Genet. 2012; 44: 825–830. 10.1038/ng.2314 22706313 PMC3386481

[30] Lipka AE, Tian F, Wang Q, Peiffer J, Li M, Bradbury PJ, et al GAPIT: genome association and prediction integrated tool. Bioinformatics. 2012; 28: 2397–2399. 22796960 10.1093/bioinformatics/bts444

[31] Stein LD, Mungall C, Shu S, Caudy M, Mangone M, Day A, et al The generic genome browser: a building block for a model organism system database. Genome Res. 2002; 12: 1599–1610. 12368253 10.1101/gr.403602 PMC187535

[32] Ravi M, Chan SW. Haploid plants produced by centromere-mediated genome elimination. Nature. 2010; 464: 615–618. 10.1038/nature08842 20336146

[33] Lynch JP. Steep, cheap and deep: an ideotype to optimize water and N acquisition by maize root systems. Ann Bot. 2013; 112: 347–357. 10.1093/aob/mcs293 23328767 PMC3698384

[34] Narang RA, Bruene A, Altmann T. Analysis of phosphate acquisition efficiency in different *Arabidopsis* accessions. Plant Physiol. 2000; 124: 1786–1799. 11115894 10.1104/pp.124.4.1786 PMC59875

[35] Svistoonoff S, Creff A, Reymond M, Sigoillot-Claude C, Ricaud L, Blanchet A, et al Root tip contact with low-phosphate media reprograms plant root architecture. Nat Genet. 2007; 39: 792–796. 17496893 10.1038/ng2041

[36] Ward JT, Lahner B, Yakubova E, Salt DE, Raghothama KG. The effect of iron on the primary root elongation of *Arabidopsis* during phosphate deficiency. Plant Physiol. 2008; 147: 1181–1191. 10.1104/pp.108.118562 18467463 PMC2442553

[37] Atwell S, Huang YS, Vilhjalmsson BJ, Willems G, Horton M, Li Y, et al Genome-wide association study of 107 phenotypes in Arabidopsis thaliana inbred lines. Nature. 2010; 465: 627–631. 10.1038/nature08800 20336072 PMC3023908

[38] Deikman J, Ulrich M. A novel cytokinin-resistant mutant of *Arabidopsis* with abbreviated shoot development. Planta. 1995; 195: 440–449. 7766046 10.1007/BF00202603

[39] Ahn SJ, Shin R, Schachtman DP. Expression of *KT/KUP* genes in *Arabidopsis* and the role of root hairs in K^+^ uptake. Plant Physiol. 2004; 134: 1135–1145. 14988478 10.1104/pp.103.034660 PMC389937

