# Supplementary material for: Uncovering Genes and Ploidy Involved in the High Diversity in Root Hair Density, Length and Response to Local Scarce Phosphate in Arabidopsis thaliana
Source: PLoS One. 2015 Mar 17;10(3):e0120604. doi: 10.1371/journal.pone.0120604 (PMC4364354; doi:10.1371/journal.pone.0120604)
Supplement: S1 Table — (DOC) [file pone.0120604.s001.doc]

| **S1_Table:** List of *Arabidopsis* accessions used in this study and assigned population name. | | | | |
| --- | --- | --- | --- | --- |
| **No** | **Ecotype** | **NASC-ID** | **Country of origin** | **Population** |
| 1 | Ag-0 | N22630 | France | Central Europe |
| 2 | Agu-1 * | N76409 | Spain | Iberian Peninsula |
| 3 | Aitba-2 * | N76347 | Tunisia | Iberian Peninsula |
| 4 | Ak-1 | N6602 | Germany | Central Europe |
| 5 | Alc-0 | N1656 | Spain | Iberian Peninsula |
| 6 | Altenb-2 * | N76353 | Italy | Mediterranean |
| 7 | Altenb-3 * |  | Italy | Mediterranean |
| 8 | An-1 | N6603 | Belgium | Central Europe |
| 9 | Angel-1 * | N76362 | Italy | Mediterranean |
| 10 | Angit * | N76366 | Italy | Mediterranean |
| 11 | Apost-1 * | N76368 | Italy | Mediterranean |
| 12 | Bay-0 | N22633 | Germany | Central Europe |
| 13 | Bil-7 | N22579 | Sweden | Northern Europe |
| 14 | Bla-1 | N970 | Spain | Iberian Peninsula |
| 15 | Bla-11 | N985 | Spain | Iberian Peninsula |
| 16 | Bolin-1 * | N76373 | Romania | Central Europe |
| 17 | Bor-1 | N22590 | Czech Republic | Central Europe |
| 18 | Bor-4 | N22591 | Czech Republic | Central Europe |
| 19 | Borsk-2 * | N76421 | Russia | Central Asia |
| 20 | Bozen-1 * | N76357 | Italy | Mediterranean |
| 21 | Bozen85 * |  | Italy | Mediterranean |
| 22 | Bur-0 | N22656 | United Kingdom | Central Europe |
| 23 | C24 | N22620 | Portugal | Iberian Peninsula |
| 24 | Can-0 | N1064 | Canary Islands | Canary Islands |
| 25 | Caste25 * |  | Italy | Mediterranean |
| 26 | Caste37 * |  | Italy | Mediterranean |
| 27 | Castelfed-4 * | N76355 | Italy | Mediterranean |
| 28 | CIBC-17 | N22603 | United Kingdom | Central Europe |
| 29 | Ciste-1 * | N76359 | Italy | Mediterranean |
| 30 | Ciste-2 * | N76360 | Italy | Mediterranean |
| 31 | Co-2 | N1086 | Portugal | Iberian Peninsula |
| 32 | Col-0 | N22625 | Poland | Central Europe |
| 33 | Copac-1 * | N76420 | Romania | Central Europe |
| 34 | Ct-1 | N6674 | Italy | Mediterranean |
| 35 | Cvi-0 | N902 | Cape Verde | Cape Verde Islands |
| 36 | Del-10 * | N28890 | Serbia | Mediterranean |
| 37 | Dobra-1 * | N76369 | Serbia | Mediterranean |
| 38 | Dog-4 * | N76386 | Turkey | Central Asia |
| 39 | Eden-1 | N22572 | Sweden | Northern Europe |
| 40 | Edi-0 | N6688 | Scotland | Central Europe |
| 41 | Est-1 | N22629 | Estonia | Northern Europe |
| 42 | Fei-0 * | N22645 | Portugal | Iberian Peninsula |
| 43 | Ga-0 | N22634 | Germany | Central Europe |
| 44 | Galdo-1 * | N76423 | Italy | Mediterranean |
| 45 | Gie-0 | N6720 | Germany | Central Europe |
| 46 | Got-22 | N22609 | Germany | Central Europe |
| 47 | Got-7 | N22608 | Germany | Central Europe |
| 48 | Gu-0 | N22617 | Germany | Central Europe |
| 49 | Guntschnna-1 * |  | Italy | Mediterranean |
| 50 | Gy-0 | N22631 | France | Central Europe |
| 51 | Hi-0 | N6736 | Netherlands | Central Europe |
| 52 | HKT2-4 * | N76404 | Germany | Central Europe |
| 53 | HR-10 | N22597 | United Kingdom | Central Europe |
| 54 | HR-5 | N22596 | United Kingdom | Central Europe |
| 55 | Jablo-1 * | N76372 | Greece | Mediterranean |
| 56 | Kidr-1 * | N76376 | Russia | Central Asia |
| 57 | Kin-0 | N22654 | USA | North America |
| 58 | Kly-1 * | N9630 | Russia | Central Asia |
| 59 | Kn-0 | N6762 | Lithuania | Northern Europe |
| 60 | Knox-10 | N22566 | USA | North America |
| 61 | Knox-18 | N22567 | USA | North America |
| 62 | Koch-1 * | N22823 | Ukraine | Central Europe |
| 63 | Kondara | N22651 | Tajikistan | Central Asia |
| 64 | Koz-2 * | N9637 | Russia | Central Asia |
| 65 | Kr-0 | N1296 | Germany | Central Europe |
| 66 | Krazo-2 * | N76422 | Russia | Central Asia |
| 67 | Kurta1441 * |  | Italy | Mediterranean |
| 68 | Kurta1532 * |  | Italy | Mediterranean |
| 69 | Kurta16313 * |  | Italy | Mediterranean |
| 70 | Kz-1 | N22606 | Kazakhstan | Central Asia |
| 71 | Kz-9 | N22607 | Kazakhstan | Central Asia |
| 72 | Laats335 * |  | Italy | Mediterranean |
| 73 | Lago-1 * | N76367 | Italy | Mediterranean |
| 74 | Leb-3 * | N9641 | Russia | Central Asia |
| 75 | Lecho-1 * | N76371 | Bulgaria | Mediterranean |
| 76 | Ler-1 | N22618 | Poland | Central Europe |
| 77 | Lerik1-3 * | N22712 | Azerbaijan | Central Asia |
| 78 | LL-0 | N22650 | Spain | Iberian Peninsula |
| 79 | Ll-1 | N1341 | Spain | Iberian Peninsula |
| 80 | Lp2-2 | N22594 | Czech Republic | Central Europe |
| 81 | Lp2-6 | N22595 | Czech Republic | Central Europe |
| 82 | Lz-0 | N22615 | France | Central Europe |
| 83 | Mammo-1 * | N76365 | Italy | Mediterranean |
| 84 | Mammo-2 * | N76364 | Italy | Mediterranean |
| 85 | Mer-6 * | N76414 | Spain | Iberian Peninsula |
| 86 | Mitt103 * |  | Italy | Mediterranean |
| 87 | Mitt113 * |  | Italy | Mediterranean |
| 88 | Mitt62212 * |  | Italy | Mediterranean |
| 89 | Mitt8324 * |  | Italy | Mediterranean |
| 90 | Mitt9311 * |  | Italy | Mediterranean |
| 91 | Mitterberg-1 * | N76354 | Italy | Mediterranean |
| 92 | Monte-1 * | N76361 | Italy | Mediterranean |
| 93 | Moran-1 * | N76363 | Italy | Mediterranean |
| 94 | Mr-0 | N1373 | Italy | Mediterranean |
| 95 | Mrk-0 | N22635 | Germany | Central Europe |
| 96 | Ms-0 | N22655 | Russia | Northern Europe |
| 97 | Mt-0 | N1380 | Libya | Mediterranean |
| 98 | Mz-0 | N22636 | Germany | Central Europe |
| 99 | N13 | N22491 | Russia | Northern Europe |
| 100 | N7 | N22485 | Russia | Northern Europe |
| 101 | Nd-1 | N22619 | Germany | Central Europe |
| 102 | NFA-8 | N22598 | United Kingdom | Central Europe |
| 103 | Nie1-2 * | N76402 | Germany | Central Europe |
| 104 | No-0 | N3081 | Germany | Central Europe |
| 105 | Oy-0 | N22658 | Norway | Northern Europe |
| 106 | Petergof | N926 | Russia | Northern Europe |
| 107 | Petro-1 * | N76370 | Serbia | Mediterranean |
| 108 | Pla-0 | N6834 | Spain | Iberian Peninsula |
| 109 | Pla-1 | N1461 | Spain | Iberian Peninsula |
| 110 | Pla-3 | N1464 | Spain | Iberian Peninsula |
| 111 | Pna-10 | N22571 | USA | North America |
| 112 | Pna-17 | N22570 | USA | North America |
| 113 | Po-0 ** | N6839 | Germany | Central Europe |
| 114 | Pu2-23 | N22593 | Czech Republic | Central Europe |
| 115 | Pu2-7 | N22592 | Czech Republic | Central Europe |
| 116 | Ra-0a | N22632 | France | Central Europe |
| 117 | Ren-1 | N22610 | France | Central Europe |
| 118 | Ren-11 | N22611 | France | Central Europe |
| 119 | Rmx-A02 | N22568 | USA | North America |
| 120 | Rovero-1 * | N76351 | Italy | Mediterranean |
| 121 | Roverod26 * |  | Italy | Mediterranean |
| 122 | RRS-10 | N22565 | USA | North America |
| 123 | RRS-7 | N22564 | USA | North America |
| 124 | Rsch-4 | N6850 | Russia | Northern Europe |
| 125 | Se-0 | N1503 | Spain | Iberian Peninsula |
| 126 | Sf-2 | N1517 | Spain | Iberian Peninsula |
| 127 | Sha * | N6180 | Tajikistan | Central Asia |
| 128 | Shigu-2 * | N76374 | Russia | Central Asia |
| 129 | Sij-1 * | N76379 | Uzbekistan | Central Asia |
| 130 | Sij-2 * | N76380 | Uzbekistan | Central Asia |
| 131 | Sij-4 * | N9656 | Uzbekistan | Central Asia |
| 132 | Slavi-1 * | N76419 | Bulgaria | Central Europe |
| 133 | Sorbo | N22653 | Tajikistan | Central Asia |
| 134 | Sq-1 | N22600 | United Kingdom | Central Europe |
| 135 | Sq-8 | N22601 | United Kingdom | Central Europe |
| 136 | Star-8 * | N76400 | Germany | Central Europe |
| 137 | Stepn-1 * | N76378 | Russia | Central Asia |
| 138 | Stepn-2 * | N76377 | Russia | Central Asia |
| 139 | Tamm-2 | N22604 | Finland | Northern Europe |
| 140 | Timpo-1 * | N76424 | Italy | Mediterranean |
| 141 | Ts-1 | N22647 | Spain | Iberian Peninsula |
| 142 | Ts-5 | N22648 | Spain | Iberian Peninsula |
| 143 | Tsu-0 | N6874 | Japan | Japan |
| 144 | Tsu-1 | N22641 | Japan | Japan |
| 145 | Tu-SB30-3 | N76403 | Germany | Central Europe |
| 146 | Ull2-3 | N22587 | Sweden | Northern Europe |
| 147 | Uod-1 | N22612 | Austria | Central Europe |
| 148 | Uod-7 | N22613 | Austria | Central Europe |
| 149 | Van-0 | N1585 | Canada | North America |
| 150 | Vash-1 * | N22754 | Georgia | Central Asia |
| 151 | Vezza63 * |  | Italy | Mediterranean |
| 152 | Vezzano-2 * | N76350 | Italy | Mediterranean |
| 153 | Voeran-1 * | N76352 | Italy | Mediterranean |
| 154 | Wa-1 | N1587 | Poland | Central Europe |
| 155 | Wal-HasB-4 * | N76408 | Germany | Central Europe |
| 156 | Wei-0 | N22622 | Switzerland | Central Europe |
| 157 | Wil-2 | N6889 | Russia | Northern Europe |
| 158 | Ws-0 | N1602 | Russia | Northern Europe |
| 159 | Wt-5 | N22637 | Germany | Central Europe |
| 160 | Wu-0 ** | N6897 | Germany | Central Europe |
| 161 | Xan-1 * | N76387 | Azerbaijan | Central Asia |
| 162 | Yeg-1 * | N76394 | Armenia | Central Asia |
| 163 | Yo-0 | N1623 | USA | North America |
| 164 | Zdr-1 | N22588 | Czech Republic | Central Europe |
| 165 | Zdr-6 | N22589 | Czech Republic | Central Europe |
| 166 | Zu-0 ** | N6902 | Germany | Central Europe |
| * Accessions used for identification of SNPs that change amino acids | | | | |
| ** Accessions not used for genome wide association mapping | | | | |
