# Supplementary figures and images for: Uncovering Genes and Ploidy Involved in the High Diversity in Root Hair Density, Length and Response to Local Scarce Phosphate in Arabidopsis thaliana
Source: PLoS One. 2015 Mar 17;10(3):e0120604. doi: 10.1371/journal.pone.0120604 (PMC4364354; doi:10.1371/journal.pone.0120604)

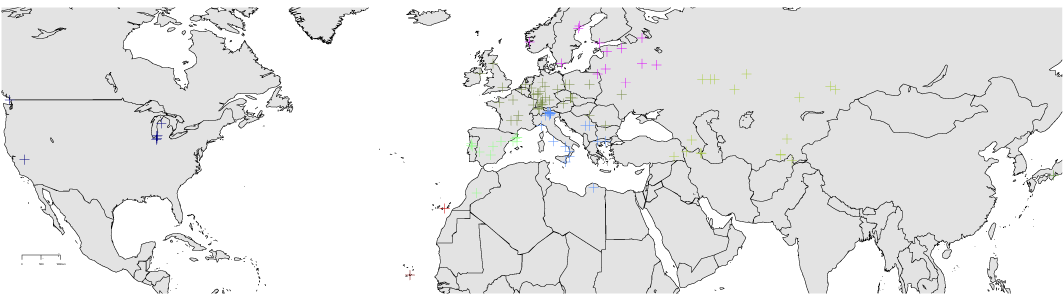

Supplement: S1 Fig — Different colors indicate regional sub-populations. (TIF) [file pone.0120604.s003.tif]

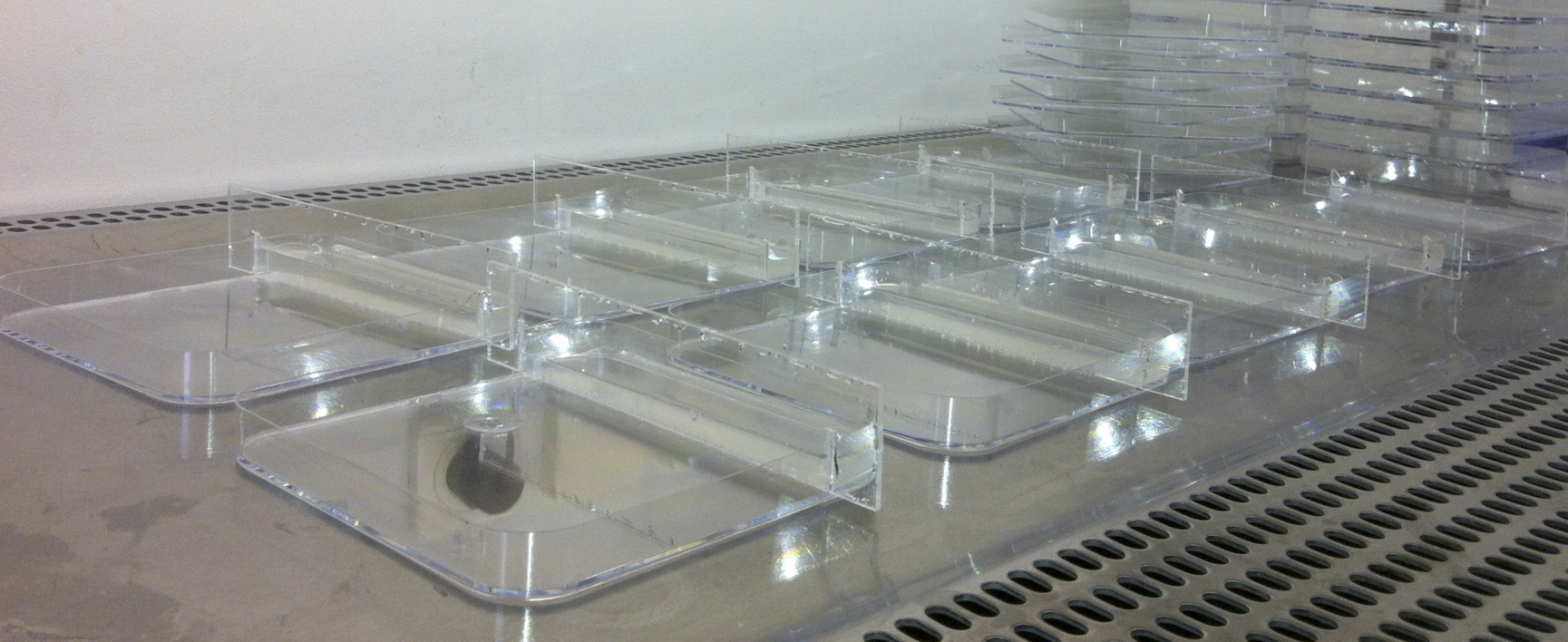

Supplement: S2 Fig — The barrier in the plates was removed after solidification of the top compartment. (TIF) [file pone.0120604.s004.tif]

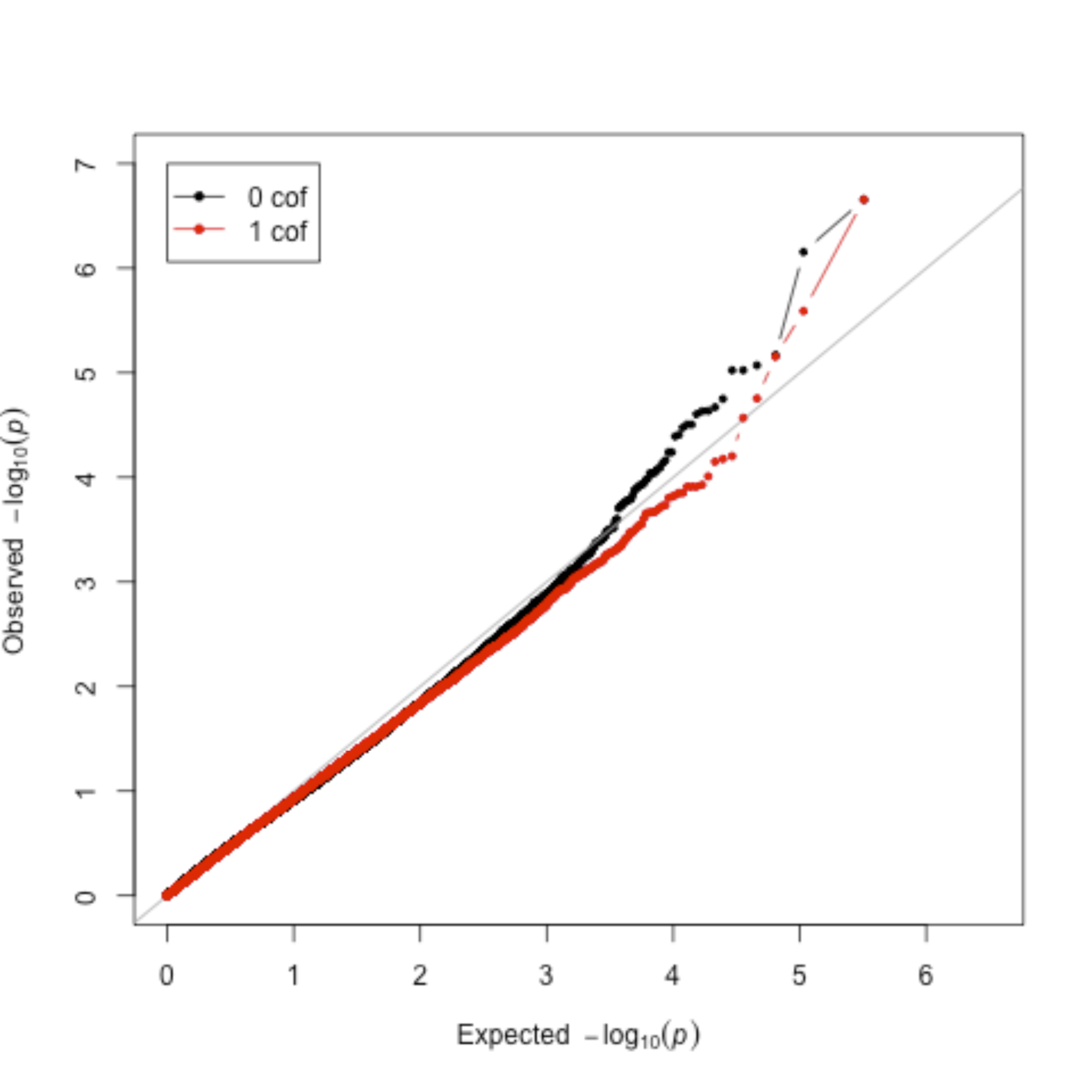

Supplement: S3 Fig — First order (black dots) and second order (red dots) corrections for observed and expected distribution due to population bias. (TIF) [file pone.0120604.s005.tif]

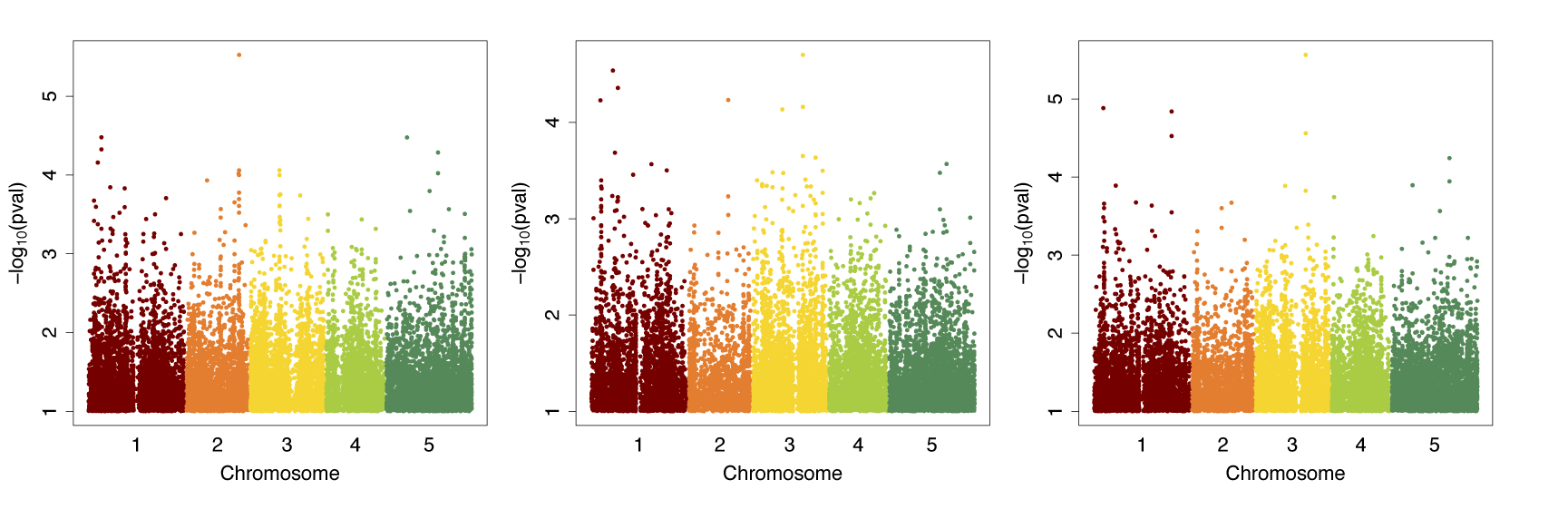

Supplement: S4 Fig — SNPs of different chromosomes are given in different colors. (TIF) [file pone.0120604.s006.tif]

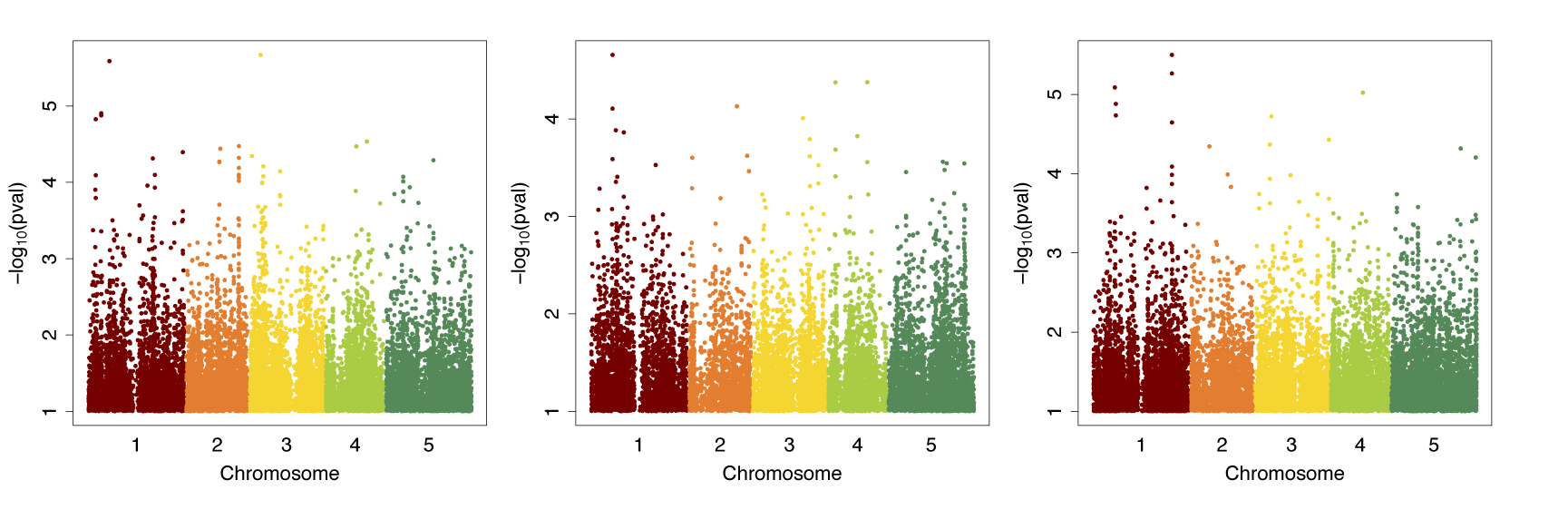

Supplement: S5 Fig — SNPs of different chromosomes are given in different colors. (TIF) [file pone.0120604.s007.tif]
